# Low Rates of MASLD Screening in Young Adults With Type 2 Diabetes: A Retrospective Cohort Study

**DOI:** 10.1210/jendso/bvaf223

**Published:** 2025-12-29

**Authors:** Chloe A de Crecy, Qiang Yang, Mei Liu, Rui Yin, Anu Sharma

**Affiliations:** Division of Endocrinology, Diabetes and Metabolism, University of Florida College of Medicine, Gainesville, FL 32610, USA; Department of Health Outcomes and Biomedical Informatics, University of Florida College of Medicine, Gainesville, FL 32610, USA; Department of Health Outcomes and Biomedical Informatics, University of Florida College of Medicine, Gainesville, FL 32610, USA; Department of Health Outcomes and Biomedical Informatics, University of Florida College of Medicine, Gainesville, FL 32610, USA; Division of Endocrinology, Diabetes and Metabolism, University of Florida College of Medicine, Gainesville, FL 32610, USA

**Keywords:** young adults, MASLD/NAFLD, diabetes mellitus type 2, screening, early detection of disease, risk assessment

## Abstract

**Context:**

Regardless of age, metabolic dysfunction-associated steatotic liver disease (MASLD) occurs in 70% of adults with type 2 diabetes (T2D) and increases the risk of metabolic dysfunction-associated steatohepatitis (MASH), fibrosis, and cirrhosis. However, many studies focus on older adults.

**Objective:**

To determine the frequency of screening for MASLD in young adults with T2D attending outpatient clinics.

**Methods:**

A retrospective cohort study on young adults (aged 18-44 years) with T2D who accessed care at a tertiary care center from January 1, 2018, to December 31, 2022. At-risk MASH was diagnosed with vibration-controlled transient elastography, magnetic resonance elastography, or liver biopsy.

**Results:**

Of 6891 participants included, 16% (n = 1100) had a diagnosis of MASLD. Those with MASLD were more likely to have cardiometabolic risk factors (83% vs 72%, *P* < .001) and cardiovascular disease (22% vs 16%, *P* < .001). Only 12% of those with MASLD underwent further investigation (1.9% of the total cohort), which was associated with dyslipidemia (odds ratio [OR], 2.4; 95% CI, 1.5-3.8), ALT >40 U/L (OR, 2.1; 95% CI, 1.4-3.0), or the use of 3 or more diabetes medications (OR, 2.1; 95% CI, 1.5-3.1). In those with further workup, 38% had at-risk MASH. The fibrosis-4 index was elevated in 16% of those with MASLD and only 28% of those with confirmed at-risk MASH or worse.

**Conclusion:**

Screening for MASLD in young adults with T2D is frequently missed. There is a lack of accurate noninvasive tools in this population. Increased awareness of screening young adults with T2D for at-risk MASH is urgently needed to prevent progression to cirrhosis.

The global surge of type 2 diabetes (T2D) in young adults is fueling a parallel rise in metabolic dysfunction–associated steatotic liver disease (MASLD), driving earlier onset and higher risks of cirrhosis and cardiometabolic complications (1). Although 70% of people with T2D have MASLD, 20% to 35% will develop metabolic-dysfunction associated steatohepatitis (MASH) with clinically significant fibrosis (fibrosis stage ≥2) or at-risk MASH—an important harbinger of cirrhosis (2, 3). In addition, MASH increases the risk of mortality, hepatocellular carcinoma (even in the absence of cirrhosis), liver transplantation, and extrahepatic cancers (4-6). As therapies now exist that slow the progression and even reverse fibrosis (7, 8), screening for at-risk MASH in people with T2D is recommended by the American Diabetes Association and other organizations (9-11). However, at-risk MASH is a tissue-based diagnosis, which poses a significant barrier to screening individuals who might be at risk. Luckily, the detection of clinically significant fibrosis on imaging (eg, vibration-controlled transient elastography [VCTE], magnetic resonance elastography [MRE]) is an acceptable and reliable surrogate to identify at-risk MASH.

Prior studies have shown that young adults with T2D are at a similarly elevated risk of developing at-risk MASH when compared to older adults (12). In fact, up to 1 in 7 young adults with T2D and obesity have hepatic fibrosis. Yet, young adults remain largely absent from MASLD screening studies (13) and from validation of noninvasive tests like the fibrosis-4 (FIB-4) index, the first step in risk stratifying adults with T2D for at-risk MASH (9, 10). As a result, the performance and real-world applicability of current risk stratification recommendations in young adults are not known. Even in older adults, MASLD screening is low: a mere 9% of adults with T2D (mean age 62 ± 14 years) with an indeterminate or high FIB-4 received further testing or subspecialty referral (14). Screening rates for MASLD among young adults with T2D, however, have not been well described. In addition, the FIB-4 is less reliable in individuals younger than age 35 years (15), with studies showing decreased accuracy even in those younger than age 45 years (16). This poses a significant barrier to reliably identifying young adults with T2D who have MASLD based on current guideline recommendations. Taken together, there is a critical need to define current MASLD screening rates and to assess how existing risk stratification recommendations for MASLD support this growing at-risk population. The aims of this study, therefore, were to determine the frequency of screening for at-risk MASH among young adults with T2D attending outpatient clinics and to evaluate the clinical utility of current MASLD risk stratification recommendations in this population.

## Materials and Methods

### Study Population

This was a retrospective cohort study using electronic health records from the University of Florida Shands Health system, including patients seen at any University of Florida Health location in Gainesville, Florida, from January 1, 2018, to December 31, 2022. Inclusion criteria were age 18 to 44 years at first diagnosis of T2D in our integrated data repository, which was started in 2012, identified by International Classification of Diseases (ICD) codes (ICD 9 codes 250.xx and ICD 10 codes E11.xx). Exclusion criteria were no history of T2D or other forms of diabetes mellitus (eg, type 1 diabetes mellitus, cystic fibrosis-related diabetes, pancreatogenic diabetes, monogenic forms of diabetes), ages <18 years or ≥45 years, diagnosis of other types of liver disease (eg, viral hepatitis, alcohol-related liver disease, autoimmune liver disease, hemochromatosis, drug-induced hepatitis, liver metastases), missing demographic data, or recorded refusal to participate in database studies. The study was approved by the University of Florida Institutional Review Board (# 202300939) and followed STROBE guidelines (Table S1) (17). The requirement for informed consent was waived because of the minimal risk associated with the study design.

**Figure 1.**
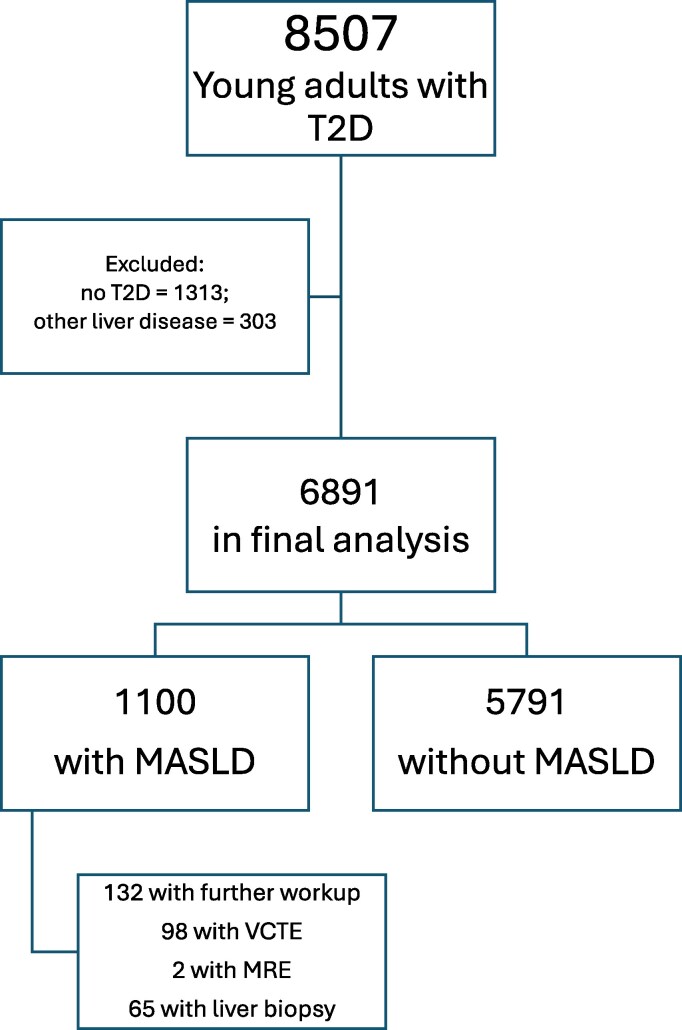
CONSORT diagram of study participants.

Of an initial cohort of 8507 young adults with T2D, 1616 participants were excluded due to lack of T2D or the presence of another type of diabetes, another cause of liver disease, or missing demographic data; a total of 6891 participants were included in the final analysis (Fig. 1).

### Study Design

In this retrospective cross-sectional analysis, participants’ electronic health records were first analyzed by computational analysis to confirm the presence of T2D (18) and to identify the absence or presence of a diagnosis of MASLD. An MASLD diagnosis was identified by ICD codes, including ICD 9 codes 571.8, 571.9, 573.8, or 573.8 and ICD 10 codes K74.00, K74.01, K74.02, K74.60, 74.69, K75.81, or K76.0. For those without an assigned diagnosis of MASLD but were evaluated by imaging for MASLD (CPT code 91200 for VCTE, CPT code 76981 for ultrasound elastography, or CPT code 76391 for MRE or liver biopsy (CPT codes 47000, 47001, 47002, 47003, 4379), the charts were reviewed in detail and assigned as MASLD present if the results were consistent with MASLD. MASLD degree was classified as steatosis, fibrosis, or cirrhosis. Any fibrosis was defined as liver stiffness measurement on VCTE (VCTE-LSM) of ≥7.0 kPA, or on MRE as LSM-MRE of ≥2.65 kPA (19), or on liver biopsy as a fibrosis stage ≥1. At-risk MASH was defined as a VCTE-LSM of ≥8.0 kPA (9), or on MRE as LSM-MRE of ≥3.14 kPA (19), or on liver biopsy as a MASLD (NAFLD) activity score of ≥4 and fibrosis stage ≥2 based on the Clinical Research Network liver pathology criteria (20).

The primary outcome was the proportion of participants with a MASLD diagnosis code who received further testing by imaging or pathology (VCTE, ultrasound-elastography, MRE, or liver biopsy) in young adults with T2D. Secondary endpoints were the accuracy of FIB-4 in risk-stratifying young adults with T2D, clinical characteristics that were associated with at-risk MASH, and the number of young adults with T2D with at-risk MASH, advanced fibrosis, or cirrhosis. The diagnosis of fibrosis or cirrhosis was determined by the results of imaging or biopsy. If more than 1 modality was performed, results of the liver biopsy was used over any other modality and MRE was used over VCTE. Patient clinical characteristics collected were at the first clinical encounter associated with T2D ICD code within the time period (not necessarily at first diagnosis, which was defined as the age first associated with a T2D ICD code that occurred before the prespecified period). Data analyzed included age at the encounter, self-reported sex, self-reported race/ethnicity, body mass index at the encounter, comorbidities (hypertension, dyslipidemia, chronic kidney disease, coronary artery disease, heart failure, cerebrovascular accident history), laboratory values including hemoglobin A1c, platelets, aspartate aminotransferase (AST), alanine aminotransferase (ALT), prescription for diabetes medications (metformin, pioglitazone, DDP4 inhibitors, sodium glucose cotransporter 2 inhibitors, GLP1R agonists), and prescription for antihypertensives and statins. Comorbidities were defined by their associated ICD 9 or 10 codes and/or use of medication to treat the disease. Only outpatient laboratory data and outpatient prescriptions were collected and analyzed.

AST to platelet ratio (APRI) was calculated as:


$$\text{APRI}=[(\text{AST in IU / L})/(\text{AST upper limit of normal in IU/L})]÷(\text{Platelets in}\;10^{9}/\text{L})$$

where the upper limit of normal for AST was 35 IU/L and an APRI >0.5 (21) was defined as indicative of at risk for hepatic fibrosis from MASLD.

FIB-4 (22) was calculated as:


$$\begin{array}{rl} \text{FIB}-4\;= & \;[(\text{Age}\;\times \;(\text{AST in IU / L})]\;÷\;[(\text{Platelets in}10^{9}/\text{L})\\ & \times \surd (\text{ALT in IU / L})] \end{array}$$

where FIB-4 ≥ 1.3 (10) was defined as indicative of at risk for hepatic fibrosis from MASLD.

### Statistical Analysis

For the final analysis, the cohort was divided into 2 groups based on the presence or absence of an associated diagnosis code for MASLD. Categorical variables were reported as percentages with group differences determined by the Pearson chi-squared or Fisher exact test when indicated. Continuous variables were reported as means and SDs when normally distributed and medians with interquartile ranges if not normally distributed. A 2-sample *t*-test or Wilcoxon rank-sum test was used to determine differences in continuous variables. Logistic regression was used to estimate univariate associations between clinical variables and odds of undergoing further investigation. Multivariable regression was then performed of the significant unadjusted variables and then removed until only the significant variables remained. Missing data were handled by data removal. *P* < .05 was considered significant. Analyses were performed using JMP Pro 18.0 and GraphPad Prism 10.6.

## Results

### Participant Characteristics

Of the 6891 young adults with T2D, 16% (n = 1100) had an associated diagnosis for MASLD. There were no differences between age, but more women were diagnosed with T2D (60% female vs 40% male) and with MASLD (59% without MASLD vs 63% with MASLD, *P* = .036; Table 1). Young adults with T2D and MASLD were more likely to have cardiometabolic risk factors (obesity, hypertension, or dyslipidemia: 72% vs 83%, *P* < .001) and cardiovascular disease (16% vs 22%, *P* < .001). In addition, young adults with T2D and MASLD were more likely to use any diabetes-related medications and statins. There were no differences in glycemic control (glycated hemoglobin 7.6 ± 2.5% without MASLD vs 7.4 ± 1.9% with MASLD, *P* = .298); however, both AST (18 [IQR 14-25] without MASLD vs 23 [16-38] U/L with MASLD, *P* < .001) and ALT (18 [IQR 12-28] without MASLD vs 27 [17-50] U/L with MASLD, *P* < .001) were significantly higher in young adults with T2D and MASLD compared to those without MASLD. Despite this, there were no differences in the calculated FIB-4 index between groups. APRI scores, however, were higher in those with MASLD compared to those without MASLD (Table 1).

**Table 1.** Participant characteristics

Clinical parameters	T2D without MASLD (n = 5791)	T2D with MASLD (n = 1100)	*P* value	Missing (n)
Age at T2D diagnosis, years	36 (7)	36 (7)	.982	0
Current age, years	40 (7)	40 (7)	.249	0
Sex (F/M), %	59/41	63/37	.036	0
Race, %			<.001	167
White	47	62		
Black	38	25		
Asian	2	3		
Other/unknown	13	10		
Hispanic, %	11	12	.161	186
Comorbidities, %				0
Hypertension	60	75	<.001	
Dyslipidemia	46	65	<.001	
CKD	10	10	.854	
CAD	9	14	<.001	
Heart failure	9	12	.005	
CVA	4	4	.925	
Deceased, %	4	4	.328	0
BMI, kg/m^2^	37 (11)	40 (10)	<.001	256
BMI categories, %			<.001	
Normal weight	10	4		
Overweight	17	12		
Obesity	73	84		
Diabetes medications, %	51	66	<.001	0
Metformin	14	16	.067	
Sulfonylureas	4	9	<.001	
Pioglitazone	8	11	<.001	
DPP4i	11	17	<.001	
SGLT2i	19	35	<.001	
GLP-1Ra	29	36	<.001	
Insulin				
≥3 noninsulin T2D medications, %	13	23	<.001	0
HTN medications, %	39	53	<.001	0
Statin, %	31	43	<.001	0
HbA1c, %	7.6 (2.5)	7.4 (1.9)	.298	2057
Platelets, ×10^9^/μL	273 (87)	269 (83)	.151	1067
AST, U/L*^a^*	18 (14-25)	23 (16-38)	<.001	1537
ALT, U/L*^a^*	18 (12-28)	27 (17-50)	<.001	1536
FIB-4 index	0.81 (0.68)	0.95 (0.95)	<.001	1725
FIB-4 ≥ 1.3, %	12	16	<.001	
APRI	0.25 (0.29)	0.37 (0.43)	<.001	1724
APRI ≥0.5, %	8	19	<.001	

Abbreviations: ALT, alanine aminotransferase; APRI, AST to platelet ratio; AST, aspartate aminotransferase; BMI, body mass index; CAD, coronary artery disease; CKD, chronic kidney disease; CVA, cerebrovascular disease; DPP4, dipeptidyl peptidase-4 inhibitor; FIB-4, fibrosis-4; GLP-1Ra, glucagon-like peptide-1 receptor agonist; HTN, hypertension; MASLD, metabolic dysfunction-associated steatotic liver disease; SGLT2i, sodium glucose cotransporter 2 inhibitor; T2D, type 2 diabetes.

^a^Results reported as median (25th-75th interquartile range).

### Investigation of At-risk MASH

**Figure 2.**
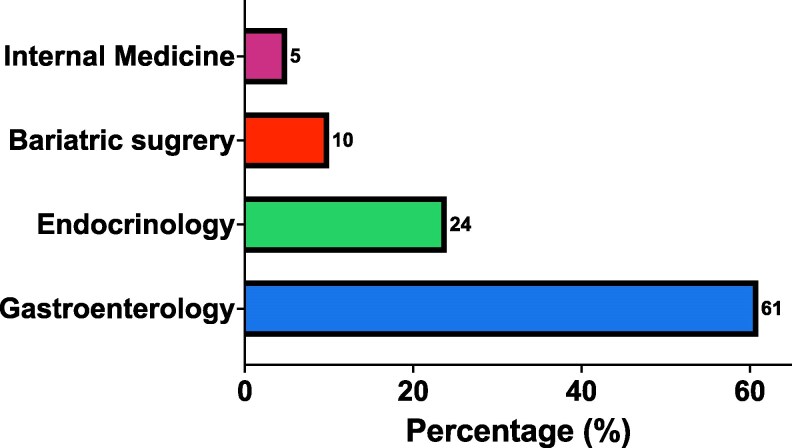
Clinics that ordered further investigation in participants with metabolic dysfunction-associated steatotic liver disease.

Of the 1100 young adults with T2D with MASLD, only 12% (n = 132) underwent further investigation with imaging or biopsy for at-risk MASH (1.9% of the total cohort). VCTE was the most used imaging (n = 98), followed by liver biopsy (n = 65) and MRE (n = 2). No participant underwent ultrasound-elastography. Of those with a liver biopsy, 50% (n = 32) underwent VCTE before biopsy and 1 person underwent VCTE before MRE. Investigation for at-risk MASH was largely ordered by gastroenterology (61%) followed by endocrinology (24%) (Fig. 2). Among young adults with T2D and MASLD, those who underwent further investigation were more likely to be older (41 ± 6 years with further investigation vs 40 ± 7 years without further investigation, *P* = .008) but there were no differences between groups in self-reported race and ethnicity (Table 2). Interestingly, those who underwent further investigation were less likely to have chronic kidney disease (5% with further investigation vs 11% without further investigation, *P* = .025) and have any major cardiovascular event (coronary artery disease, heart failure, or cerebrovascular disease) (13% with further investigation vs 24% without further investigation, *P* = .006). Finally, AST and ALT concentrations were higher in those undergoing further investigation (AST: 30 [IQR 17-43] U/L vs 23 [IQR 16-37] U/L, *P* = .032; ALT: 39 [IQR 19-64] U/L vs 26 [IQR 17-47] U/L, *P* = .002), more so in those who had concentrations above the upper limit of normal per the laboratory reference ranges (AST > 35 U/L: 36% vs 14%, *P* < .001; ALT > 40 U/L: 48% vs 16%, *P* < .001). Sex, glycated hemoglobin, FIB-4, and APRI scores were not different between those with and without further investigation.

**Table 2.** Participant characteristics of those with MASLD who underwent further investigation vs those who did not

Clinical parameters	MASLD without investigation (n = 962)	MASLD with investigation (n = 132)	*P* value
Current age, years	40 (7)	41 (6)	.008
Sex (F/M), %	62/38	65/35	.506
Race, %			.213
White	61	66	
Black	26	17	
Asian	3	5	
Other/unknown	10	12	
Hispanic, %	12	13	.795
Comorbidities, %			
Hypertension	75	79	.228
Dyslipidemia	63	80	<.001
CKD	11	5	.025
CAD	15	8	.011
Heart failure	13	5	.003
CVA	4	2	.190
MACE	24	13	.006
BMI, kg/m^2^	40 (10)	39 (8)	.348
BMI categories, %			.134
Normal weight	4	1	
Overweight	12	13	
Obesity	84	86	
Diabetes medications, %			
Metformin	64	78	.010
Sulfonylureas	15	17	.525
Pioglitazone	8	17	<.001
DPP4 inhibitors	11	17	.070
SGLT2i	16	25	.014
GLP-1Ra	33	56	<.001
Insulin	36	36	.862
≥3 noninsulin T2D medications, %	21	36	<.001
HTN medications, %	53	56	.564
Statin, %	42	52	.029
HbA1c, %	7.3 (1.9)	7.2 (1.5)	.512
Platelets, ×10^9^/μL	269 (83)	268 (87)	.915
AST, U/L*^a^*	23 (16-37)	30 (17-43)	.032
AST >35 U/L, %	27	35	.053
ALT, U/L*^a^*	26 (17-47)	39 (19-64)	.002
ALT >40 U/L, %	32	48	<.001
FIB-4 index ≥1.3, %	16	16	.963
APRI ≥0.5, %	23	32	.025

Abbreviations: ALT, alanine aminotransferase; APRI, AST to platelet ratio; AST, aspartate aminotransferase; BMI, body mass index; CAD, coronary artery disease; CKD, chronic kidney disease; CVA, cerebrovascular disease; DPP4, dipeptidyl peptidase-4 inhibitor; FIB-4, fibrosis-4; GLP-1Ra, glucagon-like peptide-1 receptor agonist; HTN, hypertension; MASLD, metabolic dysfunction-associated steatotic liver disease; SGLT2i, sodium glucose cotransporter 2 inhibitor; T2D, type 2 diabetes.

^a^Results reported as median (25th-75th interquartile range).

**Figure 3.**
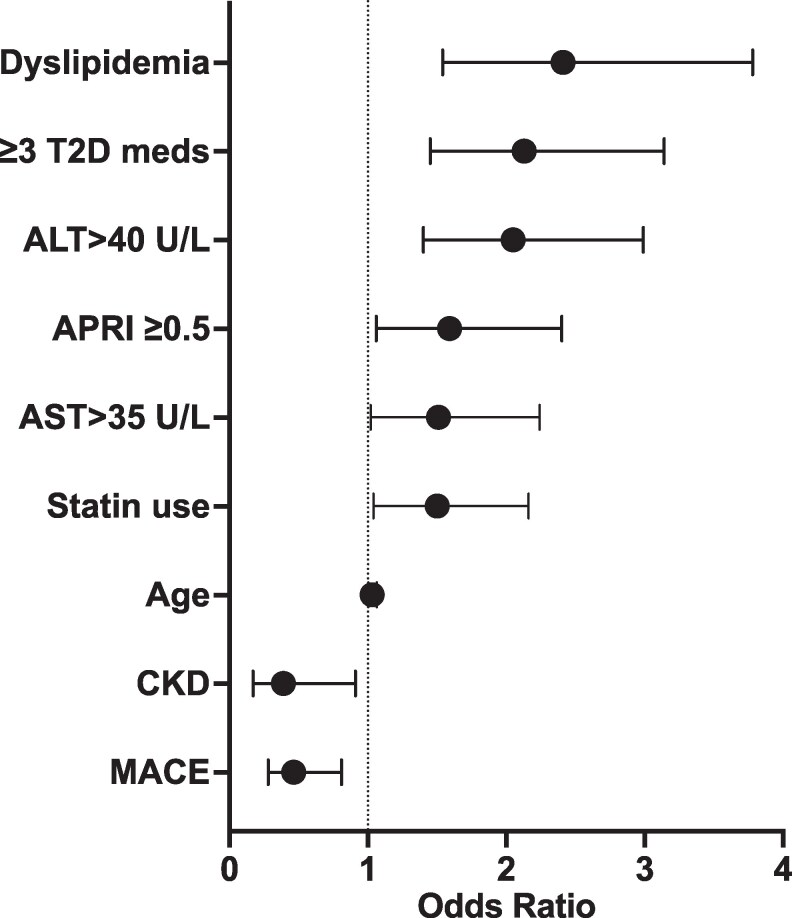
Odds ratios with univariate analysis of variables that were associated with further investigation.

After performing univariate logistic regression of the significantly different variables in Table 2, AST and ALT did not have significant calculated odds ratios for further investigation; however, AST and ALT above the upper limit of normal were significant (Fig. 3). Multivariable analysis was then performed of all the unadjusted significant variables. Only dyslipidemia remained significantly associated with further investigation for at-risk MASH (OR 2.66; 95% CI, 1.57-4.53).

### Prevalence of At-risk MASH on Further Investigation

**Figure 4.**
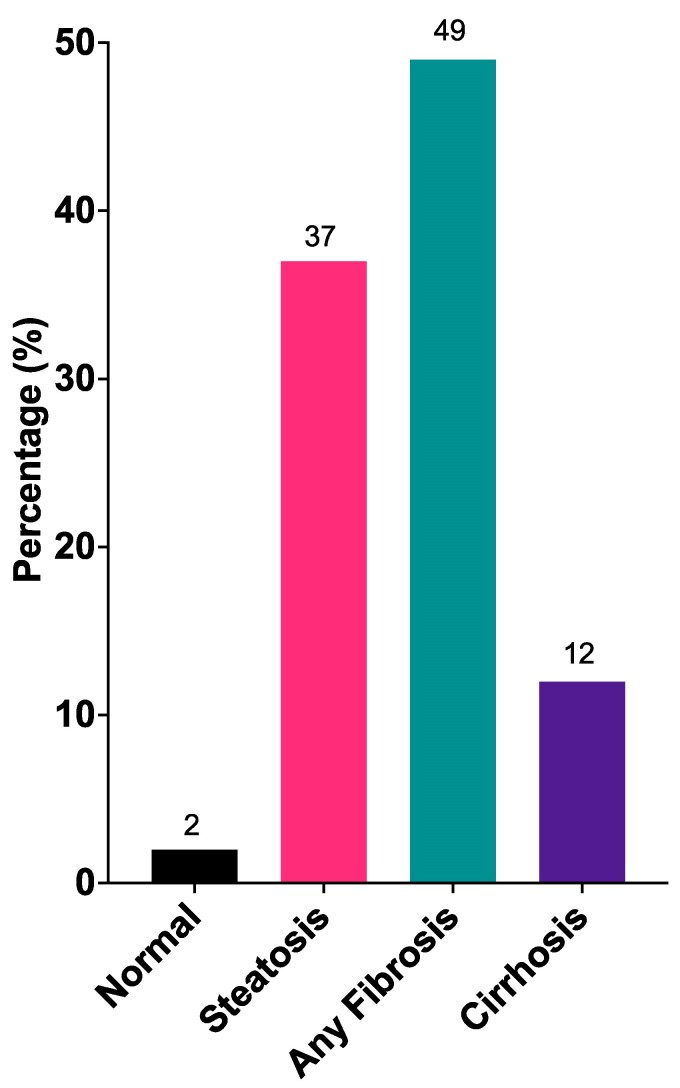
Classification of metabolic dysfunction-associated steatotic liver disease for all participants who underwent further investigation.

**Figure 5.**
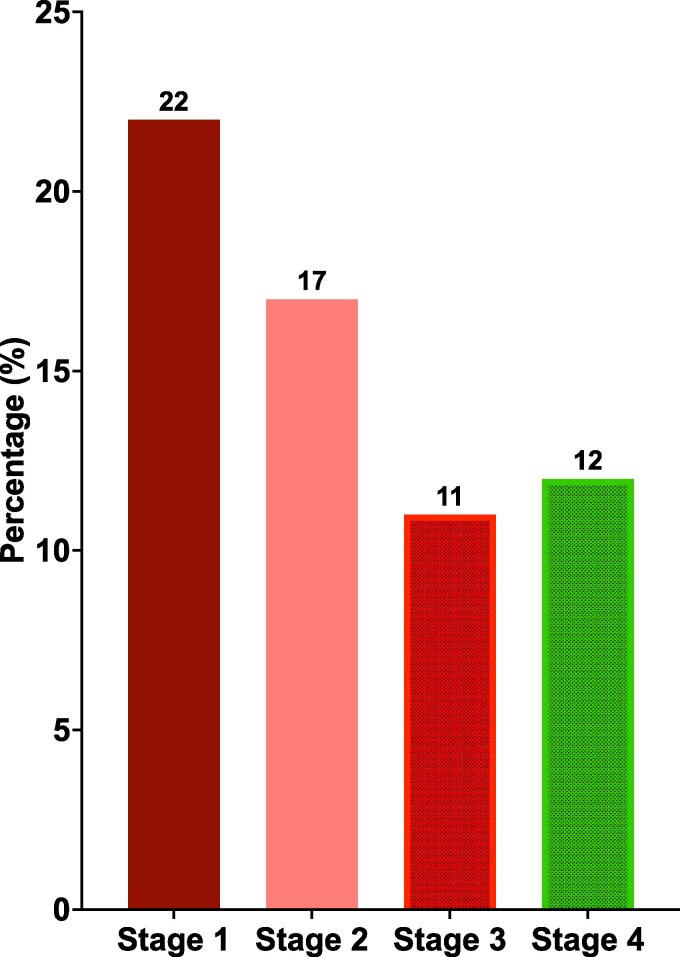
Classification of the stage of fibrosis for participants with hepatic fibrosis.

Of those who had further investigation (n = 132), 38% met criteria for at-risk MASH. Demographic variables (except self-reported race), comorbidities, body mass index (BMI), medications used, and glycemic control were not significantly different between those who had at-risk MASH compared to those who did not (Table 3). Both AST and ALT were significantly higher in those with at-risk MASH but only 51% and 58% of those with at-risk MASH had levels above the upper limit of normal, respectively. Using the previously reported sex-based ALT thresholds of >30 U/L for men and >19 U/L for women (23), 87% of those with at-risk MASH or worse had elevated ALT concentrations compared to 61% of those without at-risk MASH (*P* = .002). Most of the participants who underwent further investigation had either hepatic fibrosis or cirrhosis (Fig. 4) with 11% diagnosed with advanced fibrosis (stage 3) and 12% with cirrhosis (Fig. 5).

**Table 3.** Participant characteristics of those with vs without clinically significant fibrosis (fibrosis stage ≥2)

Clinical parameters	T2D without F ≥ 2 (n = 82)	T2D with F ≥ 2 (n = 50)	*P* value
Current age, years	41 (7)	42 (6)	.551
Sex (F/M), %	71/29	56/44	.085
Race, %			.006
White	55	84	
Black	24	6	
Asian	7	0	
Other/unknown	14	10	
Hispanic, %	12	14	.892
Comorbidities, %			
Hypertension	73	88	.043
Dyslipidemia	79	82	.702
CKD	4	6	.531
CAD	6	10	.411
Heart failure	6	2	.273
CVA	1	2	.722
MACE	13	12	.814
BMI, kg/m^2^	38 (8)	41 (7)	.059
BMI categories, %			.138
Normal weight	1	0	
Overweight	17	6	
Obesity	82	94	
Diabetes medications, %			
Metformin	73	81	.290
Sulfonylurea	15	22	.279
Pioglitazone	20	13	.298
DPP4 inhibitors	16	19	.677
SGLT2i	21	30	.280
GLP-1Ra	51	64	.168
Insulin	35	36	.894
≥3 noninsulin T2D medications, %	34	40	.498
HTN medication, %	49	66	.054
Statin, %	55	51	.668
HbA1c, %	7.5 (1.8)	7.3 (1.2)	.627
Platelets, ×10^9^/μL	284 (79)	241 (94)	.017
AST, U/L*^a^*	21 (16-36)	36 (26-58)	.002
AST >35 U/L, %	26	51	.005
ALT, U/L*^a^*	30 (16-64)	45 (33-67)	.011
ALT >40 U/L, %	43	58	.115
CAP ≥274 dB/m, %	85	91	.404
LSM, kPa	6.5 (2.5)	15.4 (7.9)	<.001
Liver biopsy, %	43	60	.054
FIB-4 index ≥1.3, %	8	28	.002
APRI ≥0.5, %	22	48	.003

Abbreviations: ALT, alanine aminotransferase; APRI, AST to platelet ratio; AST, aspartate aminotransferase; BMI, body mass index; CAD, coronary artery disease; CKD, chronic kidney disease; CVA, cerebrovascular disease; DPP4, dipeptidyl peptidase-4 inhibitor; FIB-4, fibrosis-4; GLP-1Ra, glucagon-like peptide-1 receptor agonist; HTN, hypertension; MASLD, metabolic dysfunction-associated steatotic liver disease; SGLT2i, sodium glucose cotransporter 2 inhibitor; T2D, type 2 diabetes.

^a^Results reported as median (25th-75th interquartile range).

### Subgroup Analysis in Age >30 Years Not on Insulin

A subgroup analysis was performed in those greater than age 30 years and not on insulin (n = 4150) to confirm reliability of results in a cohort of people with T2D. Similar to the total cohort, 17% had a diagnosis code for MASLD, with 12% of those with MASLD undergoing further investigation (1.7% of this subgroup). There were no differences in age, sex, or BMI among those who underwent further workup compared to those who did not. However, hypertension (78% vs 57%, *P* = .001), dyslipidemia (75% vs 37%, *P* < .001), and higher concentrations of AST (30 [IQR 17-41] U/L vs 19 [IQR 14-27] U/L, *P* < .001) and ALT (40 [IQR 17-68] U/L vs 20 [IQR 13-32] U/L, *P* < .001) were more common in those who underwent further workup compared to those who did not. Of those who underwent further investigation, 36% had at-risk MASH.

### Application of the Risk Stratification Pathway to Young Adults with Type 2 Diabetes

If the MASLD risk-stratification pathway were applied to the cohort, 25% (n = 1725) would not enter the algorithm because not all the laboratory variables were present to calculate the FIB-4. When variables for the FIB-4 were present (n = 5166), 13% (n = 646) had a FIB-4 ≥ 1.3 and should have undergone further investigation. Of these, 3% (n = 19) had further imaging or liver biopsy. Given that confirmation was performed in only 1.9% of the cohort, we applied the risk stratification pathway to this subgroup. In this subgroup (n = 132), 15% (n = 19) had a FIB-4 ≥ 1.3% and 7% (n = 9) did not have all the variables present to calculate the FIB-4. The FIB-4 correctly classified 11% as at-risk MASH (true positives) and 58% as absence of at-risk MASH (true negatives) but incorrectly classified 5% as at-risk MASH (false positive) and 27% as absence of at-risk MASH (false negative). The FIB-4's sensitivity was 28.3% and specificity was 92.2%.

## Discussion

In this study, we performed a large-scale assessment of MASLD screening in young adults with T2D and show that MASLD is underrecognized and that the current fibrosis risk-stratification algorithm using the FIB-4 is poorly suited for young adults with T2D. Among 6891 young adults with T2D, only 16% had an associated diagnosis code for MASLD and only 12% of those with a MASLD diagnosis code (1.9% of the total cohort) underwent further risk assessment with imaging or with a liver biopsy despite 13% of the cohort meeting criteria for screening based on a FIB-4 ≥ 1.3. This highlights a large care gap in the management of young adults with T2D. Cardiometabolic risk factors were prevalent among young adults with T2D. Most young adults in our cohort were afflicted with obesity and hypertension. Despite higher liver transaminases among young adults with MASLD, the FIB-4 was elevated in only 16% with MASLD and 28% with confirmed at-risk MASH. In addition, more than one third of young adults with T2D who underwent liver-related imaging or biopsy had at-risk MASH and 23% were diagnosed with advanced fibrosis or cirrhosis. Taken together, MASLD is a clinically significant complication in young adults with T2D that is common but underevaluated.

Previous data show that MASLD affects most adults with T2D. In an in-depth cross-sectional study where adults attending outpatient primary care and endocrinology clinics were screened with VCTE, 71% of young adults with T2D had MASLD (12); however, we found only 16% of young adults with T2D were diagnosed with MASLD. On top of this, only 1.9% of the entire cohort underwent further evaluation for MASLD. This is lower than what was seen in older adults where less than 10% were screened for MASLD (14) and in another study only 1.5% had transaminases measured (24). Several reasons can account for this care gap. First, it can be due to lack of awareness by clinicians on the need to screen all adults with T2D for MASLD, which has been highlighted in several prior studies. In a survey and qualitative interview study, primary care and endocrinology clinicians in an urban health care setting in the United States (n = 109) reported lack of knowledge on screening and 65% reported not using the recommended noninvasive tests (eg, FIB-4) to screen for MASLD (25). Similarly, in an international survey, 68% of health care respondents reported lack of awareness as the reason for not implementing MASLD screening (26). These studies are consistent with our results, where most of the further investigation was conducted by gastroenterology and not by primary care or endocrinology.

The American Diabetes Association and all major guidelines recommend a 2-tiered approach for fibrosis risk-stratification—FIB-4 followed by VCTE if the FIB-4 was ≥1.3 (9-11, 27, 28)—which has demonstrated good performance in prospective studies (negative predictive value 81% and positive predictive value 74%) (13). However, most young adults were not included (the age for inclusion started at 40 years). As mentioned earlier, the FIB-4 is less reliable in those aged <35 years and (15) and has decreased accuracy even in those aged <45 years (16, 29). Although APRI is less affected by age (16), it was developed for viral hepatitis (30) and performs poorly for MASLD prognostication. This is consistent with our results, where only 28% and 48% of proven at-risk MASH would have been risk-stratified as high risk if either the FIB-4 or APRI were used, respectively. Sensitivity of the FIB-4 to risk-stratify at-risk MASH was low in young adults with T2D in our cohort, much lower than reported in the older population (sensitivity 79.5% in adults with T2D who were a mean age of 60 years compared to a sensitivity of 28% in young adults) (31). However, one quarter of young adults with T2D in our cohort did not have the laboratory data needed for risk stratification. This gap, combined with the reduced accuracy of FIB-4 at younger ages, likely contributed to low screening rates. The American Diabetes Association Standards of Care started including the measurement of the complete blood count as part of the comprehensive medical evaluation of the patient with T2D in 2023 (32). This may explain why many patients were missing variables required to calculate the FIB-4. The use of electronic medical record based clinical care pathways can potentially fill this gap by alerting the provider within the visit that laboratory orders should be placed (if missing) or further screening or referral to hepatology is required if deemed high risk. In addition, machine learning algorithms now have the potential to be embedded into electronic health records to determine those at high risk. Current studies show that they outperform current noninvasive tools; however, more research is needed for validation across populations (33, 34).

Current consensus recommends a test with high negative predictive value for advanced fibrosis as the first step in risk stratification (9). However, in our cohort, the negative predictive value was only 68.3% for the FIB-4 to risk-stratify at-risk MASH, similar to prior studies evaluating the FIB-4 threshold of 1.3 for at-risk MASH or stage 2 fibrosis (31, 35). As such, a noninvasive biomarker with higher accuracy for at-risk MASH and that is easily accessible to clinicians in primary care and endocrinology is needed to risk stratify young adults with T2D. Some potential options would be the metabolic dysfunction-associated fibrosis-5 (MAF-5) score or fibrotic NASH index (FNI). In a comparative analysis of multiple noninvasive tests to detect at-risk MASH or clinically significant fibrosis, in young adults aged 18-35 years MAF-5 had a high sensitivity to detect both at-risk MASH and clinically significant fibrosis (92% and 71%, respectively), and the FNI score had a sensitivity of 88% for at-risk MASH but only 40% for clinically significant fibrosis (36). However, the MAF-5 uses the measurement of waist circumference, which is not currently routinely measured in clinics and the FNI performed poorly for clinically significant fibrosis. The NIS2+ (noninvasive score 2) is a validated blood-based biomarker for at-risk MASH (37). Compared to multiple other noninvasive blood-based tests, including FIB-4 and APRI, the diagnostic accuracy NIS2+ was consistent across multiple age ranges and thus can be a useful noninvasive biomarker in young adults (16, 37). Age-adjusted recommendations to risk-stratify young adults are desperately needed to prevent missed opportunities for treatment and current recommendations should be modified to include the currently available tools that perform superiorly in young adults.

Young adults with T2D have a higher burden of cardiometabolic risk factors, increasing their risk for future cardiovascular disease and mortality. Those with MASLD were more likely to have hypertension, obesity, dyslipidemia, and cardiovascular disease. This is consistent with the current literature in which MASLD increases cardiovascular risk and mortality (38) and is part of the cardiometabolic continuum (9, 39). A younger age at diagnosis of T2D is also associated with higher cardiovascular burden and future risk of diabetes-related complications (40). An early start to these comorbidities represents a longer lifetime need for health care and increased economic costs (41). In fact, MASLD confers a 42% incremental increased cost to T2D (42). Despite their higher risk for advanced disease, young adults with T2D who had cardiovascular disease were less likely to undergo further investigation in our cohort but those who required a more complex diabetes regimen (3 or more noninsulin medications) or who had elevated liver transaminases were more likely to undergo further investigation, particularly through gastroenterology. This highlights the fallacy that elevated liver enzymes predict hepatic fibrosis and a likely underlying bias to investigate the more medically complex but without already known cardiovascular disease. Liver transaminases are poor predictors of at-risk MASH because laboratory-based normative ranges are inaccurate and require age- and sex-adjusted thresholds (43). Although medical complexity may increase the risk of MASLD, further investigation was missed in 87% of young adults who would have benefited from an earlier diagnosis of at-risk MASH. This missed opportunity will lead to a delay in diagnosis and a higher risk of developing more advanced disease (ie, cirrhosis). Recent advances have led to the approval semaglutide (44) and resmetirom (7) for MASH fibrosis. These medications can halt or even reverse moderate-to-advanced fibrosis (stage 2-3), thereby preventing future cirrhosis. Early identification of at-risk MASH is essential to enable timely therapeutic interventions aimed at preventing or delaying morbidity and mortality.

This study was a large, well-defined cohort using real-word data to determine care patterns in young adults with T2D. When available, objective data with laboratory, imaging, and pathology results were reviewed and included. In addition, secondary chart review by clinicians trained in MASLD to confirm at-risk MASH was performed to improve accuracy of the diagnosis. However, the inherent retrospective design with the reliance on diagnosis codes introduces bias. MASLD is commonly undercoded, which likely underestimated the true prevalence of disease. In addition, many young adults had codes for both type 1 diabetes and T2D. Some may have been inaccurately excluded from the study due to a type 1 diabetes code. Similarly, most participants did not have results for c-peptide or type 1 diabetes antibodies to verify accurate classification of T2D. Accurate data on alcohol intake, socioeconomic factors, insurance coverage, and genetic data were not available to study. Some participants may have declined further evaluation or sought care at other facilities that were not captured in our study. Laboratory reference ranges may have changed over time and concomitant AST, ALT, and platelets were not available in all participants. Finally, there were limited participants who underwent further investigation so true prevalence of at-risk MASH in young adults in T2D could not be determined.

In summary, young adults with T2D are infrequently screened for at-risk MASH despite having a comparable risk to older adults. Current noninvasive tools for risk-stratification perform suboptimally in young adults with T2D and too often fail to detect at-risk MASH. There is an urgent need to develop and validate age-specific noninvasive strategies to detect at-risk MASH in young adults. Current guideline recommendations should be adjusted to either recommend VCTE in young adults with T2D if available as the first step in screening or include noninvasive tests with higher accuracy than the FIB-4 in young adults (eg, MAF-5, FNI, NIS2+). Future research should focus on developing noninvasive tools that are accurate across age groups and have a high sensitivity to screen for at-risk MASH. The use of machine-learning algorithms that are embedded into clinical workflows to predict at-risk MASH are likely future solutions to improve screening and diagnosis of at-risk MASH, but they require more in-depth validation before adoption. Finally, understanding and developing methods to overcome barriers to screening within primary care and endocrinology settings are essential to facilitate earlier diagnosis and intervention, ultimately reducing progression to cirrhosis in this high-risk group.

## Data Availability

The data that support the findings of this study are available from the authors, but restrictions apply to the availability of these data, which were used under license from the University of Florida for the current study and so are not publicly available. Data are, however, available from the authors upon reasonable request and with permission from the University of Florida.

## Abbreviations

ALT: alanine aminotransferase
APRI: AST to platelet ratio
AST: aspartate aminotransferase
BMI: body mass index
FIB-4: fibrosis-4
FNI: fibrotic NASH index
ICD: International Classification of Diseases
LSM: liver stiffness measure
MAF-5: metabolic dysfunction-associated fibrosis-5
MASH: metabolic-dysfunction associated steatohepatitis
MASLD: metabolic dysfunction-associated steatotic liver disease
MRE: magnetic resonance elastography
T2D: type 2 diabetes
VCTE: vibration-controlled transient elastography
